# Effect of Strabismus Surgery on Meibomian Glands, Ocular Surface Parameters, and Conjunctival Impression Cytology

**DOI:** 10.3390/diagnostics15101291

**Published:** 2025-05-21

**Authors:** Şenol Sabancı, Canan Sadullahoğlu, Sibel Yavuz, Mehmet Fatih Küçük, Esra Kızıldağ Özbay, Muhammet Kazım Erol, Berna Doğan

**Affiliations:** 1 Department of Ophthalmology, University of Health Science, Antalya Education and Research Hospital, 07100 Antalya, Turkey; drsibelakyol@hotmail.com (S.Y.); drmfkucuk@gmail.com (M.F.K.); esrakizildag.md@gmail.com (E.K.Ö.); muhammetkazimerol@gmail.com (M.K.E.); bernadoga3@hotmail.com (B.D.); 2 Department of Pathology, University of Health Science, Antalya Education and Research Hospital, 07100 Antalya, Turkey; canan-rana@hotmail.com

**Keywords:** Strabismus surgery, impression cytology, ocular surface parameters, dry eye

## Abstract

**Background/Objectives**: To investigate the effect of strabismus surgery on ocular surface parameters, meibomian glands, and conjunctival impression cytology. **Methods**: Preoperative and postoperative (10th day, first month, and third month) tear break-up time (TBUT) tests, Schirmer 1 tests, corneal staining scores (CSS), meibomian gland (MG) loss rates, ocular surface disease index (OSDI) scores, and conjunctival impression cytology (IC) results of 30 patients who underwent strabismus surgery were compared. **Results**: Significant differences were found between preoperative TBUT test results and those evaluated on the postoperative 10th day and at the postoperative first month (*p* < 0.0001 for both). There were also significant differences between the preoperative and postoperative first- and third-month Schirmer 1 test results (*p* = 0.02 and *p* < 0.0001, respectively). Furthermore, mean OSDI scores significantly differed between preoperative and postoperative 10th-day measurements (*p* < 0.0001). The mean postoperative 10th-day CSS was found to be significantly higher than the preoperative mean CSS (*p* < 0.0001). The stages in preoperative conjunctival IC samples were found to be significantly lower than those evaluated at all postoperative times (*p* < 0.0001 for all). Significant differences were observed between the preoperative lower eyelid MG loss rate and all postoperative MG loss rates (*p* < 0.0001 for the 10th day and first month and *p* < 0.001 for the third month). Lastly, the preoperative upper eyelid MG loss rate significantly differed from all postoperative MG loss rates (*p* < 0.0001 for the 10th day, *p* < 0.003 for the first month, and *p* < 0.0001 for the third month). **Conclusions**: We observed changes indicative of dry eye in the mean OSDI score, TBUT, Schirmer 1 test, MG loss rates, and conjunctival IC findings up to the postoperative third month in patients who underwent strabismus surgery. Therefore, we believe that patients undergoing strabismus surgery should be followed up for ocular surface diseases, particularly dry eye.

## 1. Introduction

Strabismus is the loss of parallelism between the two eyes. Although it usually occurs in childhood, it can develop at any age. A previous study reported a strabismus prevalence of 2.1% to 3.3% among children aged 6–71 months, depending on race [1]. Another study found that the lifetime incidence of new-onset strabismus in individuals over 19 years old was 4% [2]. While treatment strategies for strabismus vary based on its type and characteristics, the most common approaches include surgery, optical correction, and orthoptic therapy. The goal of strabismus surgery is to restore ocular parallelism, achieve motor and sensory fusion, establish binocular single vision, and improve stereopsis [3].

Many studies have examined how ocular surgeries affect the ocular surface, with dry eye reported as the most common iatrogenic condition following such procedures [4]. Dry eye significantly impacts the quality of life and can lead to dissatisfaction with surgery outcomes. Cataract surgery and corneal refractive surgery are the most commonly performed procedures known to cause dry eye [5,6]. Ocular surface diseases present with a variety of symptoms, the most common of which include foreign body sensation, stinging, tearing, redness, and itching. Several methods are used to diagnose dry eye, including the tear break-up time (TBUT) test, the Schirmer 1 test, ocular surface staining, tear osmolarity, and the ocular surface disease index (OSDI).

TBUT is a widely used test that assesses tear film stability on the cornea and can be affected by aqueous tear deficiency, meibomian gland (MG) dysfunction, and mucin layer abnormalities [7]. While recent studies report varying cut-offs, TBUT values below 10 s are generally considered indicative of dry eye [8]. The Schirmer 1 test, an easy and effective method primarily used to evaluate basal tear secretion, has also been shown to be affected by evaporative dry eye disease, with values below 5 mm suggestive of dry eye [9,10]. The OSDI score consists of 12 questions that evaluate the frequency of ocular surface symptoms and their occurrence in different environments and activities. It has been reported that higher OSDI scores correlate with greater ocular surface discomfort [11].

Conjunctival impression cytology (IC) is a technique that allows cytological examination and grading of cells in the conjunctival epithelium using cellulose acetate filter papers, first described by Egbert et al. in 1977 [12]. Meibography is an imaging method that visualizes MGs using infrared light, providing insight into the lipid layer of the tear film [13].

Strabismus surgery is one of the most commonly performed ocular procedures across all age groups, particularly in children [14]. However, there is a lack of literature evaluating the effects of strabismus surgery on the ocular surface. In the current study, we assessed and compared preoperative and postoperative meibography findings, Schirmer 1 test results, OSDI scores, corneal staining scores (CSS), and conjunctival IC findings in patients undergoing strabismus surgery.

## 2. Method

This study was approved by the Ethics Committee of Antalya Education and Research Hospital under approval number 16/23. Signed consent forms were obtained from all participants. The inclusion criteria were having not previously undergone ocular surgery, having no history of chronic topical medication use, having no symptomatic dry eyes, not receiving dry eye treatment, having a Schirmer test result of 5 mm or more, having no history of MG disease or atopy, having no history of medication use that could lead to MG dysfunction (antiandrogens, antihistamines, and isotretinoin), having no history of systemic disease, being not older than 50 years, being able to comply with the tests, having one eye undergo horizontal muscle recession and resection due to strabismus and having not complication that caused the operation prolonged

Schirmer 1 tests, TBUT tests, CSS, MG loss rate, conjunctival IC tests, and OSDI scores were evaluated preoperatively and postoperatively (10th day, first month, and third month).

For the TBUT test, a 1 mg sodium fluorescein-impregnated strip (OptiGlo, Wizcure Pharma, Gurugram, India) was wetted with artificial tears and applied to the lower conjunctival fornix. The patient was asked to blink to ensure even fluorescein distribution across the cornea. The biomicroscope light was adjusted to a cobalt blue filter, and the patient was instructed not to blink after the last blink. The time until the first black spot appeared on the cornea was recorded. This process was repeated five times, and the average of the obtained values was recorded as TBUT.

For the Schirmer test, after administering 0.5% proparacaine hydrochloride drops (Alcaine, S.A. Alcon-Couvreur N.V., Puurs, Belgium) for topical anesthesia and drying excess tears, a Schirmer filter paper strip (Optitech, Prayagraj, India) was folded and placed at the outer one-third of the lower eyelid, ensuring that it did not touch the cornea. The patient was asked to close their eyes for five minutes. At the end of this period, the wet length on the paper was measured from the folded point and recorded in millimeters.

**Figure 1:**
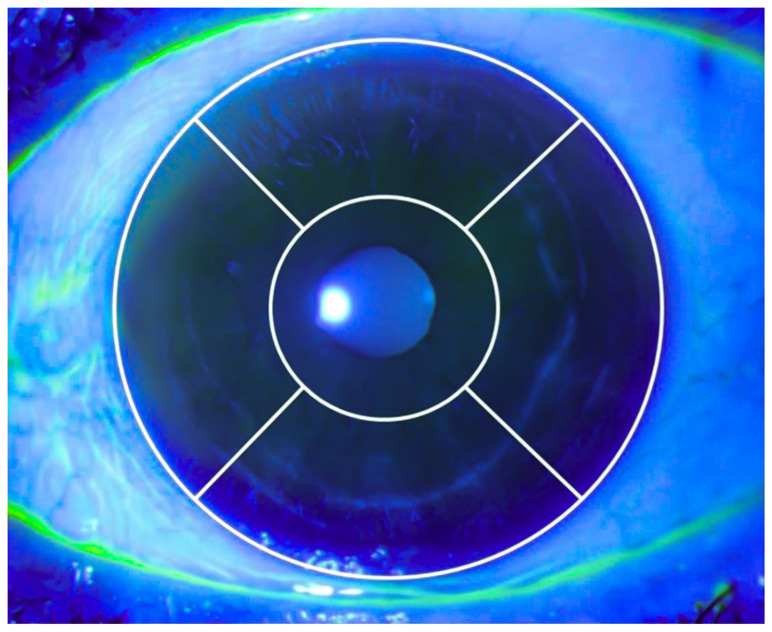
Corneal staining score calculation based on the division of the cornea into five regions: central, inferior, superior, nasal, and temporal, according to the National Eye Institute grading scale.

The CSS of each case was assessed using the National Eye Institute grading scale (Figure 1). Each cornea was divided into five equal regions: central, inferior, superior, nasal, and temporal. According to the number of punctate stains, each region was classified as follows: no staining (0 points), mild staining (1 point for 1–15 punctate stains), moderate staining (2 points for 16–30 punctate stains), and severe staining (3 points for ≥30 punctate stains). The points from all five regions were summed to obtain the total staining score for each cornea [15].

For conjunctival IC, 0.5% proparacaine hydrochloride drops (Alcaine, S.A. Alcon-Couvreur N.V., Puurs, Belgium) were applied for topical anesthesia. After drying excess tears, a 3 × 3 mm cellulose acetate filter paper (Sartorius Stedim Biotech, Göttingen, Germany) was placed on the conjunctiva 1 mm behind the superotemporal limbus, corresponding to the radial conjunctival incision site, for 10 s. The filter paper was then removed and placed in vials containing a 96% alcohol solution for fixation. These vials were stored at +4 °C to prevent alcohol evaporation. All samples were stained with periodic acid-Schiff (PAS) and hematoxylin-eosin (H&E). According to the Nelson staging method, the morphology of epithelial cells and the goblet cell count were assessed under a light microscope using a 40× objective [16]. The stages were classified as follows:

Grade 0: Epithelial cells are small and round and have large basophilic nuclei with eosinophilic cytoplasm. The nucleocytoplasmic ratio is 1/2. Goblet cells are dense, full, oval, and PAS-positive.

Grade 1: Epithelial cells are slightly larger and polygonal and have eosinophilic-staining cytoplasm. The nucleus is slightly smaller, with a nucleocytoplasmic ratio of 1/3. The number of goblet cells is reduced but still present, full, and oval, with PAS-positive cytoplasm.

Grade 2: Epithelial cells are larger and more polygonal than in Grade 1, with ample cytoplasm of variable staining. Occasionally, multinucleated cells are present. The nucleocytoplasmic ratio is 1/4–1/5. Goblet cells are significantly reduced in number and smaller, with indistinct cellular boundaries and weaker PAS-positive staining.

Grade 3: Epithelial cells are large and polygonal, with small, pyknotic nuclei or absent nuclei in most cells. Cytoplasm stains basophilically. The nucleocytoplasmic ratio is greater than 1/6. Goblet cells are completely absent.

In the evaluation of the OSDI score, each response is assigned a score between 0 (none of the time) and 4 (all of the time) based on the frequency of symptoms. The scores of all responses are summed. The OSDI score is then calculated using the following formula:

OSDI score = [(total number of points given for responses to questions × 100)/(number of questions answered) × 4]

Currently, meibography is the only method for the morphological imaging of MGs and for comparing images obtained during follow-up. In this study, non-contact meibography images were obtained using the Phoenix V.2.6 imaging program in the Sirius corneal topography device (CSO, Florence, Italy), which employs infrared light. The upper and lower eyelids of all cases were everted for imaging. Three measurements were taken for each case, and the best image was used for analysis.

**Figure 2:**
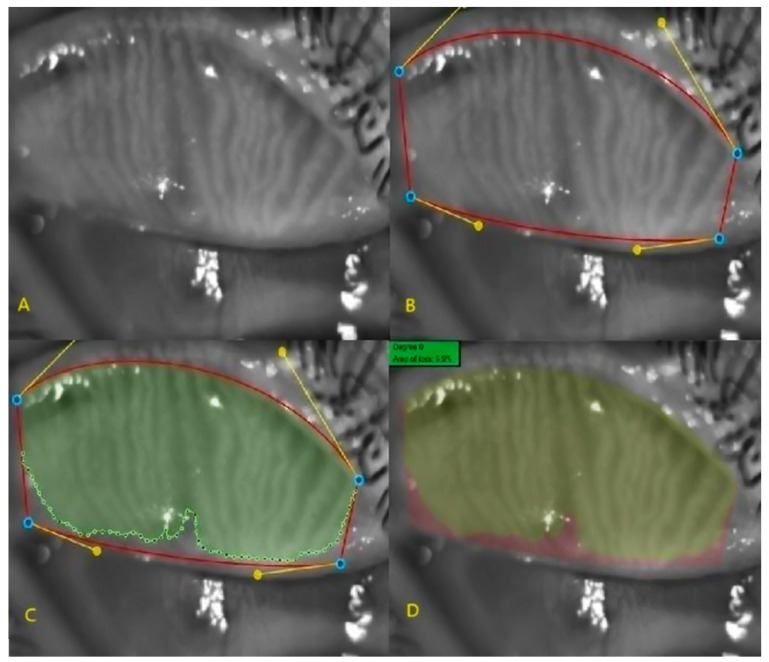
Upper eyelid meibography images: (**A**) infrared meibography image, (**B**) determination of the tarsal borders (red line), (**C**) determination of the meibomian gland borders (green area), and (**D**) determination of the meibomian gland loss area and ratio (pink area).

On the selected image, the tarsal borders were marked using the Phoenix meibography program. The MG region within these borders was then identified, and the MG loss rate was automatically calculated as a percentage (Figure 2).

Recession-resection surgery was performed under general anesthesia on one eye of each patient by the same surgeon (Ş.S.) due to strabismus. At the beginning of the operation, 5% povidone-iodine was applied to the ocular surface and left for three minutes. A 4.0 silk suture (Trusilk, Bengaluru, India) was placed on the sclera 1 mm behind the lower and upper limbus to allow eye manipulation during surgery. In all cases, the conjunctiva was incised approximately 7 mm from top to bottom, 1 mm behind the limbus in the nasal and temporal regions, with additional 4 mm radial conjunctival incisions made superiorly and inferiorly. After exposing the medial and lateral rectus muscles using strabismus hooks, the intermuscular membranes and Tenon’s capsule tissue within the surgical field were removed. For muscle recession, a 6.0 Vicryl suture (Trusynth, Bengaluru, India) was first passed through the upper and lower parts of the insertion area, initially in a lamellar fashion and then full-thickness. The suture was then secured with four knots in alternating directions. The muscle was then cut as close as possible to the insertion site and sutured to the sclera using the same suture at the designated recession distance from the insertion point. For muscle resection, the resection amount was measured from the insertion area both superiorly and inferiorly and marked on the muscle. As in the recession procedure, a 6.0 Vicryl suture was used to fix the muscle at the marked areas, and the muscle was resected anterior to the sutures. The resected muscle was then sutured to the insertion site. The conjunctival tissue was closed using an 8.0 Vicryl suture (Mitsu™, Gujarat, India). At the end of the surgery, 20 mg/mL gentamicin (Genta^®^ 20 mg/mL) and 8 mg/2 mL dexamethasone (Dekort^®^ 8 mg/2 mL) were administered to the nasal and temporal subconjunctival areas in all cases. Postoperatively, all patients were prescribed topical netilmicin drops (Netira, SIFI S.P.A., Catania, Italy) four times daily for one week and topical prednisolone acetate drops (Pred Forte, Allergan Pharmaceuticals, Mayo, Ireland) five times daily. The prednisolone acetate 1% drops were tapered weekly and discontinued after five weeks.

## 3. Statistical Method

Descriptive statistics were presented as percentages, means, standard deviations, medians, minimums, maximums, and interquartile ranges (25th percentile [Q1]–75th percentile [Q3]. The normality assumption was checked using the Shapiro-Wilk test. To compare measurements taken at four different time points, analysis of variance for repeated measures was used for variables that met the normality assumption, followed by post hoc pairwise comparisons with Bonferroni correction. The Friedman test was used for variables that did not meet the normality assumption, followed by pairwise comparisons using the Bonferroni-Dunn procedure. Statistical analyses were performed using SPSS v. 23.0, with *p*-values < 0.05 considered statistically significant.

## 4. Results

Of the 30 patients included in the study, 46.7% were female and 53.3% were male. The mean age of the patients was 26.33 ± 13.79 (6–50) years. Exotropia surgery was performed in 16 patients (53.3%), while esotropia surgery was performed in 14 patients (47.7%). Table 1 presents a comparison of preoperative and postoperative (10th day, first month, and third month) TBUT, Schirmer 1 test values, and OSDI scores. Significant differences were found between preoperative TBUT values and postoperative TBUT values at all evaluation times (*p* < 0.0001 for all). Preoperative Schirmer 1 test values were significantly higher than those at all postoperative time points, except for the 10th day (*p* < 0.05). There were also statistically significant differences in the time-dependent measurements of OSDI scores (*p* < 0.0001). The preoperative OSDI score was significantly lower than the postoperative 10th-day OSDI score (*p* < 0.0001).

The MG loss rate in both the lower and upper eyelids was significantly lower at all postoperative evaluation times compared to the preoperative period (*p* < 0.0001 for all) (Table 2).

**Figure 3:**
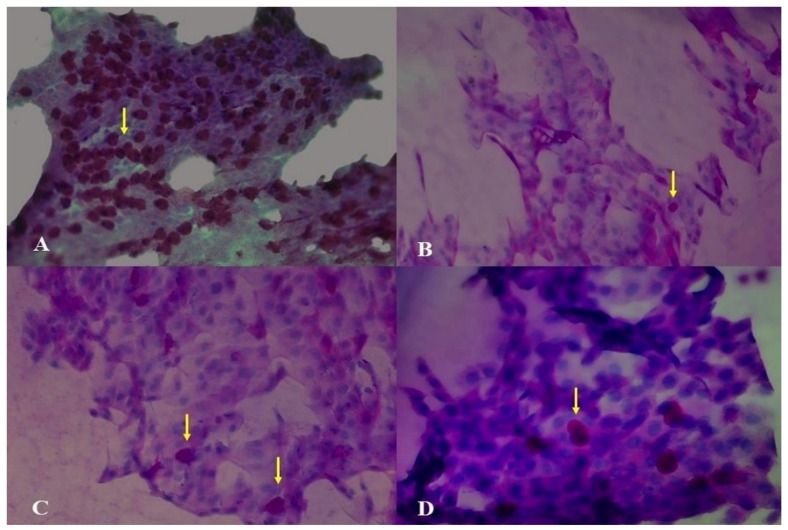
Conjunctival impression cytology images of a patient who underwent strabismus surgery: (**A**) preoperative (grade 0), (**B**) postoperative day 10 (grade 2), (**C**) postoperative month 1 (grade 2), and (**D**) postoperative month 3 (grade 1) (yellow arrows indicate goblet cells).

In addition, statistically significant differences were found in the time-dependent measurements of IC (*p* < 0.0001). IC grades were significantly higher at all postoperative evaluation times compared to preoperative values (Table 3) (Figure 3). Statistically significant differences were also observed between the preoperative CSS and the postoperative 10th-day and first-month measurements. The CSS was significantly higher in the postoperative period compared to the preoperative period (*p* < 0.0001) (Table 3).

## 5. Discussion

Ocular surface disease occurs when the conjunctiva, lacrimal gland, MG, or cornea is affected, either together or separately, and most commonly manifests as dry eye syndrome [17]. The number of ocular surgeries is increasing each year due to global population growth. It has been reported that ocular surgeries contribute to ocular surface diseases through multiple mechanisms [18]. Although the effects of cataract surgery, corneal refractive surgery, vitrectomy, and eyelid surgery on the ocular surface have been extensively studied, there is limited research on the effects of strabismus surgery [19,20,21,22].

TBUT specifically reflects the function of the mucin layer of the tear film. Mucin is secreted by intraepithelial goblet cells in the conjunctiva [23]. Conjunctival incisions made during strabismus surgery may result in a decrease in goblet cell density, reducing mucin secretion and compromising tear film stability, ultimately leading to a decrease in TBUT. The cornea receives sensory innervation via the ophthalmic branch of the trigeminal nerve, with sensory nerves entering from the limbus [24]. Conjunctival incisions and limbal manipulations during surgery may damage corneal nerves. This may reduce the blink reflex, decreasing tear secretion and increasing evaporation, which could contribute to a reduction in TBUT.

In a previous study, Li et al. found that TBUT in patients who underwent limbal conjunctival incision strabismus surgery was significantly lower at the first, second, and fourth postoperative weeks compared to preoperative values but returned to normal levels by the eighth week [25]. Similarly, Jeon et al. reported that TBUT decreased significantly in the first week after lateral rectus recession surgery using a fornix incision and increased up to four months postoperatively but did not reach preoperative values [26]. In our study, TBUT was significantly lower than preoperative values on the 10th day and at the first and third months postoperatively. Studies have also reported that conjunctival goblet cell loss leads to squamous metaplasia [27]. Consistent with these findings, we observed squamous metaplasia in all cases in all IC samples collected postoperatively, supporting the presence of goblet cell loss.

Li et al. and Chang et al. reported no significant difference between preoperative and postoperative Schirmer 1 test measurements [25,27]. In contrast, our study demonstrated a statistically significant difference between preoperative Schirmer 1 test values and all postoperative values, except for the measurement taken on the 10th postoperative day. By the third postoperative month, although the mean Schirmer test result value remained above 10 mm but it was still lower than the mean preoperative value. Possible reasons for the differences between our study and the study conducted by Li et al. include their patients’ younger mean age, the shorter waiting time after instillation of the topical anesthetic agent, and the performance of the test before the natural tear film had fully stabilized. Regarding the study undertaken by Chang et al., patients underwent different types of strabismus surgery, which may have influenced the results.

In our study, OSDI scores were significantly higher on the 10th postoperative day and remained elevated at the first postoperative month. Potential reasons for this increase include irritation from conjunctival sutures, conjunctival edema, the use of topical medications, and corneal toxicity resulting from exposure to light during surgery. This is further supported by the significant difference in CSS between the preoperative period and the 10th postoperative day and first postoperative month, which may have contributed to the elevated OSDI scores during these time points. By the third postoperative month, there was no significant difference between OSDI scores and preoperative values. This may be due to the absorption of sutures, discontinuation of topical medications, and a decrease in squamous metaplasia. Li et al. similarly reported that OSDI scores at the first, second, and fourth postoperative weeks were higher than preoperative values but found no significant difference between OSDI scores at the postoperative eighth week and preoperative values [25]. Jeon et al. also reported that McMonnies Dry Eye Questionnaire scores in patients who underwent lateral rectus recession surgery were significantly higher in the first three postoperative months but returned to preoperative values by the fourth month [26].

MGs produce the lipid layer of the tear film, which prevents tear evaporation and contributes to tear stability. Several studies have examined the effects of ocular surgery on MGs [28,29]. It has been reported that inflammation caused by surgical trauma, eyelid retractors, and postoperative topical medications may be responsible for these effects [30]. In a study conducted by Wang et al., no significant difference was found between the preoperative and postoperative meiboscore values in children who underwent strabismus surgery [31]. This may be due to the meiboscore measuring MG loss within a certain range or the fact that all cases involved children. In our study, we determined that MG loss was significantly higher in both the lower and upper eyelids at all postoperative evaluation times compared to preoperative values. The particularly high MG loss rate on the 10th postoperative day may be attributed to palpebral conjunctival edema obscuring some MGs in meibography, creating a false impression of loss. The observed increase in MG loss may contribute to the significant TBUT, CSS, Schirmer 1 test, and OSDI score results, indicating dry eye following strabismus surgery.

IC is a frequently used, non-invasive method for the cytological analysis of the ocular surface and the grading of squamous metaplasia. In the current study, all cases preoperatively had an IC grade of 0. Postoperatively, particularly on the 10th day, stage values increased, indicating squamous metaplasia in all cases. By the third postoperative month, squamous metaplasia had decreased in most cases, but none returned to preoperative levels. Squamous metaplasia is an indicator of goblet cell loss and has been reported in numerous studies [32]. In our cases, conjunctival incisions and surgical manipulations, surgery-induced inflammation, and preservatives in topical medications may have contributed to conjunctival squamous metaplasia. Kim et al. reported that mechanical irritation of the conjunctiva caused squamous metaplasia and decreased goblet cell density, which is consistent with our findings [33]. The statistically significant decrease in squamous metaplasia from the first to the third postoperative month may be attributed to the use of topical steroids. Oh et al. found that conjunctival goblet cell loss was highest on the first day after cataract surgery, and, although goblet cell density increased at the first and third postoperative months, it did not return to preoperative levels [34], which is consistent with our results.

Corneal staining is one of the symptoms of ocular surface disease and plays an important role in the diagnosis of dry eye. Ocular surgeries, particularly cataract and corneal refractive surgery, can cause corneal staining [35]. In a study by Li et al., corneal fluorescein staining scores were highest in the first postoperative week after strabismus surgery, returning to preoperative values at the fourth and eighth postoperative weeks [25]. Consistent with that study, we observed the highest CSS on the 10th postoperative day. By the third postoperative month, the CSS had decreased, and although it remained higher than the preoperative value, the difference was no longer statistically significant. Increased conjunctival squamous metaplasia, MG involvement, accessory lacrimal gland dysfunction, topical medications, phototoxicity, and ocular inflammation following strabismus surgery may cause corneal fluorescein staining. It has also been reported that limbal conjunctival incisions may damage corneal sensory nerves, playing a role in dry eye development and corneal staining [36]. It has been reported that 5% povidone-iodine used for prophylactic purposes before intravitreal injection causes damage to the corneal epithelium, and corneal staining and tear film layer abnormalities are increased in these eyes compared to the other eyes [37]. The 5% povidone-iodine that we use before strabismus surgery may also cause corneal staining and dry eyes in the early postoperative period. The decrease in the CSS at the third postoperative month may be due to the reduction in topical medication use, ocular surface inflammation, and squamous metaplasia.

The location of the initial incision in strabismus surgery may also affect the severity of ocular surface changes. The study by Li et al. reported that limbus incision strabismus surgery caused more severe dry eye symptoms and tear film abnormalities than fornix incision strabismus surgery [25]. In our study, all cases were operated with limbal incision method. Especially in the early periods, significant changes were detected in ocular surface parameters, which is consistent with this study.

Limitations of our study include the lack of long-term follow-up and the inability to measure additional objective tear parameters, such as non-invasive TBUT, tear osmolarity, tear meniscus height, and separate tear film layer measurements.

In conclusion, we observed that limbal conjunctival incision strabismus surgery could cause temporary changes in the ocular surface, particularly in the first 10 postoperative days. Although most of these changes significantly regressed by the third postoperative month, they did not return to baseline levels. We consider that further studies with larger sample sizes, longer follow-up periods, and more comprehensive ocular surface assessments are needed to better understand the effects of strabismus surgery on the ocular surface.

## Figures and Tables

**Table 1:** Analysis of time-dependent changes in TBUT, Schirmer 1, and OSDI variables.

	Mean ± SD (Min–Max)	Median (Q1–Q3)	*p*	Pairwise Comparison
Preoperative TBUT	11.37 ± 2.09 (4.00–16.00)	11.00 (10.00–12.00)	<0.0001 *	*p* < 0.0001 *p*_2_ < 0.0001 *p*_3_ = 0.001 *p*_4_ = 0.99 *p*_5_ = 0.969 *p*_6_ = 0.99
Postoperative TBUT, day 10	7.47 ± 2.75 (2.00–14.00)	7.00 (6.00–9.00)		
Postoperative TBUT, month 1	8.50 ± 2.90 (2.00–15.00)	9.00 (6.00–11.00)		
Postoperative TBUT, month 3	8.20 ± 3.01 (3.00–14.00)	8.00 (6.00–11.00)		
Preoperative Schirmer 1 test	15.13 ± 5.42 (5.00–30.00)	14.00 (11.00–18.00)	<0.0001 **	*p*_1_ = 0.46 *p*_2_ = 0.02 *p*_3_ < 0.0001 *p*_4_ = 0.487 *p*_5_ = 0.057 *p*_6_ = 0.99
Postoperative Schirmer 1 test, day 10	12.67 ± 5.66 (2.00–30.00)	12.00 (10.00–16.00)		
Preoperative Schirmer 1 test, month 1	11.20 ± 4.07 (2.00–21.00)	10.50 (9.00–14.00)		
Preoperative Schirmer 1 test, month 3	10.50 ± 4.96 (2.00–23.00)	11.00 (8.00–13.00)		
Preoperative OSDI	25.35 ± 9.41 (11.40–52.10)	25.00 (20.80–31.30)	<0.0001 *	*p*_1_ < 0.0001 *p*_2_ < 0.0001
Postoperative OSDI, day 10	54.36 ± 11.05 (34.10–78.10)	54.60 (46.90–62.50)		*p*_3_ = 0.113 *p*_4_ = 0.113
Postoperative OSDI, month 1	45.39 ± 9.20 (31.30–62.50)	43.60 (37.50–52.10)		*p*_5_ < 0.0001 *p*_6_ = 0.008
Postoperative OSDI, month 3	35.47 ± 8.94 (17.90–68.20)	31.56 (31.30–39.58)		

* Friedman test, ** Repeated-measures analysis of variance. SD: standard deviation, TBUT: tear break-up time, OSDI: ocular surface disease index, *p*_1_: preoperative vs. postoperative day 10, *p*_2_: preoperative vs. postoperative month 1, *p*_3_: preoperative vs. postoperative month 3, *p*_4_: postoperative day 10 vs. month 1, *p*_5_: postoperative day 10 vs. month 3, *p*_6_: postoperative month 1 vs. month 3.

**Table 2:** Analysis of time-dependent changes in MG loss rates in the lower and upper eyelids.

MG Loss	Mean ± SD (Min–Max)	Median (Q1–Q3)	*p* *	Pairwise Comparison
Lower eyelid, preoperative	10.62 ± 11.58 (1.00–57.60)	7.45 (2.90–14.40)	<0.0001	*p* < 0.0001 *p*_2_ < 0.0001 *p*_3_ < 0.0001 *p*_4_ = 0.99 *p*_5_ = 0.99 *p*_6_ = 0.99
Lower eyelid, postoperative day 10	24.10 ± 17.80 (4.00–78.50)	17.25 (13.90–27.20)		
Lower eyelid, postoperative month 1	22.36 ± 16.13 (4.80–70.80)	17.30 (11.90–26.60)		
Lower eyelid, postoperative month 3	23.18 ± 17.05 (4.10–71.20)	20.00 (14.50–25.30)		
Upper eyelid, preoperative	13.46 ± 12.17 (2.30–50.50)	8.70 (5.00–21.40)	<0.0001	*p*_1_ < 0.0001 *p*_2_ = 0.003 *p*_3_ < 0.0001 *p*_4_ = 0.658 *p*_5_ = 0.99 *p*_6_ = 0.99
Upper eyelid, postoperative day 10	32.42 ± 19.20 (3.70–72.00)	28.25 (15.90–45.20)		
Upper eyelid, postoperative month 1	23.32 ± 13.07 (3.80–58.50)	20.20 (13.50–28.60)		
Upper eyelid, postoperative month 3	22.38 ± 12.33 (5.00–60.10)	20.65 (13.90–26.40)		

* Friedman test. SD: standard deviation, TBUT: tear break-up time, MG: meibomian gland, *p*_1_: preoperative vs. postoperative day 10, *p*_2_: preoperative vs. postoperative month 1, *p*_3_: preoperative vs. postoperative month 3, *p*_4_: postoperative day 10 vs. month 1, *p*_5_: postoperative day 10 vs. month 3, *p*_6_: postoperative month 1 vs. month 3.

**Table 3:** Analysis of the changes in CSS and IC variables over time.

	Median (Q1–Q3)	*p* *	Pairwise Comparison
CSS, preoperative	0.00 (0.00–1.00)	<0.0001	*p* < 0.0001, *p*_2_ < 0.0001, *p*_3_ = 0.273, *p*_4_ = 0.098, *p*_5_ < 0.0001, *p*_6_ = 0.042
CSS, postoperative day 10	4.00 (3.00–5.00)		
CSS, postoperative month 1	2.50 (2.00–3.00)		
CSS, postoperative month 3	1.00 (0.00–3.00)		
IC, preoperative	0.00 (0.00–0.00)	<0.0001	*p*_1_ < 0.0001, *p*_2_ < 0.0001, *p*_3_ < 0.0001, *p*_4_ = 0.727, *p*_5_ = 0.010, *p*_6_ = 0.658
IC, postoperative day 10	2.00 (2.00–2.00)		
IC, postoperative month 1	2.00 (1.00–2.00)		
IC, postoperative month 3	1.00 (1.00–1.00)		

* Friedman test. *p*_1_: preoperative vs. postoperative day 10, *p*_2_: preoperative vs. postoperative month 1, *p*_3_: preoperative vs. postoperative month 3, *p*_4_: postoperative day 10 vs. month 1, *p*_5_: postoperative day 10 vs. month 3, *p*_6_: postoperative month 1 vs. month 3.

## Data Availability

The original contributions presented in this study are included in the article. Further inquiries can be directed to the corresponding author.
